# Modular literature review: a novel systematic search and review method to support priority setting in health policy and practice

**DOI:** 10.1186/s12874-021-01463-y

**Published:** 2021-11-27

**Authors:** Annariina M. Koivu, Patricia J. Hunter, Pieta Näsänen-Gilmore, Yvonne Muthiani, Jaana Isojärvi, Pia Pörtfors, Ulla Ashorn, Per Ashorn

**Affiliations:** 1 Tampere University, FI-33014 Tampere, Finland; 2 UCL Great Ormond Street Institute of Child Health, 30 Guilford Street, London, WC1N 1EH UK; 3 Finnish Institute for Health and Welfare, FI-00271 Helsinki, Finland

**Keywords:** Modular review, Systematic review, Review methodology, Priority-setting, Health policy, Evidence-based policy, Randomised controlled trial, Effect size

## Abstract

**Background:**

There is an unmet need for review methods to support priority-setting, policy-making and strategic planning when a wide variety of interventions from differing disciplines may have the potential to impact a health outcome of interest. This article describes a Modular Literature Review, a novel systematic search and review method that employs systematic search strategies together with a hierarchy-based appraisal and synthesis of the resulting evidence.

**Methods:**

We designed the Modular Review to examine the effects of 43 interventions on a health problem of global significance. Using the PICOS (Population, Intervention, Comparison, Outcome, Study design) framework, we developed a single four-module search template in which population, comparison and outcome modules were the same for each search and the intervention module was different for each of the 43 interventions. A series of literature searches were performed in five databases, followed by screening, extraction and analysis of data. “ES documents”, source documents for effect size (ES) estimates, were systematically identified based on a hierarchy of evidence. The evidence was categorised according to the likely effect on the outcome and presented in a standardised format with quantitative effect estimates, meta-analyses and narrative reporting. We compared the Modular Review to other review methods in health research for its strengths and limitations.

**Results:**

The Modular Review method was used to review the impact of 46 antenatal interventions on four specified birth outcomes within 12 months. A total of 61,279 records were found; 35,244 were screened by title-abstract. Six thousand two hundred seventy-two full articles were reviewed against the inclusion criteria resulting in 365 eligible articles.

**Conclusions:**

The Modular Review preserves principles that have traditionally been important to systematic reviews but can address multiple research questions simultaneously. The result is an accessible, reliable answer to the question of “*what works*?”. Thus, it is a well-suited literature review method to support prioritisation, decisions and planning to implement an agenda for health improvement.

**Supplementary Information:**

The online version contains supplementary material available at 10.1186/s12874-021-01463-y.

## Background

To advance evidence-informed planning, practitioners need to know what works. In the context of limited resources, they need to decide which interventions to prioritise. Priority setting in health policy is an evidence-informed complex process considered a valuable approach to support achieving national health goals particularly in low- and middle-income countries (LMIC) [1]. It is based on underlying aims of epidemiological impact and cost-effectiveness. Additionally, decision-makers need to take into consideration aspects related to practical feasibility, balance of benefits and harms, as well as rights, equity, acceptability and other societal and health system considerations [2].

The effectiveness of interventions is a crucial part of priority setting and should be based on best available evidence. However, in most areas of healthcare, there is too much potentially relevant research for those involved in health policy or care provision to integrate in decision making [3]. A single bibliographic database can contain more than 25 million references [4]. Systematic reviews respond to this need by summarising primary research on a particular research question in a single document, using explicit, traditionally quantitative methods. However, they require a significant amount of time and effort and work best when addressing a very focused question [5] in fields where a well-developed evidence base exists [6]. Furthermore, neither original research nor traditional systematic reviews will easily find evidence gaps [7].

During the last decade, systematic reviews have been accompanied by an increasing number of other review types and methodological approaches [8]. Rapid reviews have been developed to respond to time pressures [9, 10] and umbrella reviews for the growing number of systematic reviews [11]. This reflects a paradigm shift where the traditional role of reviews - mapping research activity and consolidating existing knowledge - has been expanded to include a more pragmatic role in knowledge translation for advancing professional practice [8]. One of the more iterative and flexible approaches is the scoping review [12]. Scoping reviews are particularly suited for answering broad questions, clarifying working definitions and conceptual boundaries of a topic and exposing knowledge gaps [13]. In recent years, an updated methodological guidance for scoping reviews has been established and Preferred Reporting Items for Systematic Reviews and Meta-Analyses (PRISMA) has been developed to include scoping reviews (the PRISMA-ScR) [13–15]. However, the implementation of the method is often still considered less standardised than in systematic reviews; the heterogeneity and volume of the included literature can limit effective synthesis; and they do not generally perform a risk of bias assessments or provide intervention effect estimates which makes them unsuitable for clinical recommendations [7, 16–18].

Exploration of the effectiveness of interventions to inform priority setting represents a typical situation that requires a potentially high number of systematic, concurrent searches in a broad evidence base to find the most reliable and up-to-date data on not one but several interventions. At the same time, there is often a limited timeline to produce a synthesis that facilitates comparative discussion on multiple interventions. Yet, no method for obtaining, reviewing and synthesising data has been proposed for this purpose. In response, this paper introduces the Modular Literature Review (hereafter: Modular Review), a novel systematic search and review method for expanding current methodologies to capture quantitative estimates of effect size where available and to identify gaps and frontiers to inform research and implementation. The method was developed in response to the need to prioritise interventions to reduce the prevalence of an important global health problem.

Specifically, the aims of the project were to 1) obtain a set of data in order to consider a wide variety of interventions that potentially reduce unfavourable outcomes of interest, 2) provide estimates of effect on the outcomes of interest for each intervention where possible, 3) systematically obtain an assessment of the quality of evidence of each intervention’s effect on the outcomes of interest, 4) classify the evidence based on a balanced assessment of the strength, quality and quantity of evidence to produce an accessible synthesis and 5) achieve the above aims in 12 months.

In this paper, we describe the method developed to meet these aims and assess it in terms of the sensitivity of the searches and the time and resource usage. We compare it to other established review methods in health research that could have been utilised in the project, namely systematic reviews, scoping reviews, overviews of systematic reviews and rapid reviews, to demonstrate its value and unique contribution.

## Methods

### Context

In 2019, an international group of 16 experts working in research, clinical and funding roles in broad areas of nutrition, infection control and maternal and child health in LMIC convened a workshop to develop a common framework for action to tackle low birth weight (LBW) and its dual contributors: preterm birth (PTB) and fetal growth restriction (FGR). LBW is an important determinant of child survival and development and a large burden of morbidity and mortality may be prevented by addressing the associated risk factors [19]. The primary aim of the workshop was to initiate an expert opinion process on the best approaches to reduce LBW globally, particularly in sub-Saharan Africa and South Asia.

The mechanisms leading to LBW via PTB and FGR are complex, with many opportunities to intervene to avert poor outcomes. The expert group agreed on 43 potential interventions and sought evidence of effectiveness for each from randomised controlled trials (RCTs). Each intervention was conceptualised as a combination of a risk factor and a method of eliminating or reducing exposure to the risk factor or mitigating its impact (Additional file 1).

To respond to this sizeable research need, we developed the Modular Review method to review literature on intervention effectiveness using a hierarchical organisation of the evidence. Our core working group consisted of four researchers (AK, PH, PNG, YM), two information specialists (JI, PP), two statisticians and six research assistants. Three other information specialists supported the core team by occasional assistance in information retrieval. The team was accountable through regular meetings to two senior researchers (UA, PA). The majority of the team members worked on the project on a part-time basis.

In the autumn of 2020, a panel of international experts in global maternal and newborn health convened to review the results of the project. The group consisted of 51 experts from Africa, Asia, Europe and North America. They represented academia, governmental and non-governmental organisations, the World Health Organization, the United Nations and research funders.

### Development of the literature search

**Fig. 1:**
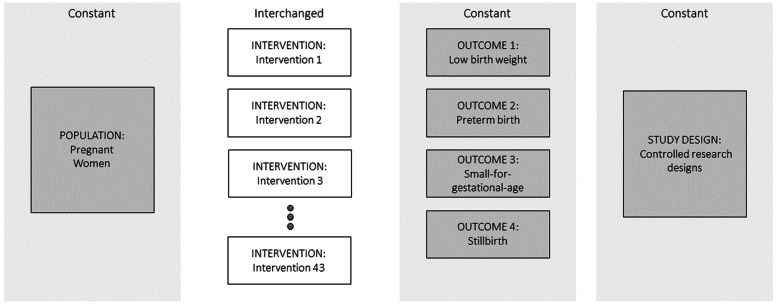
A modular PICOS-based literature search model

**Fig. 2:**
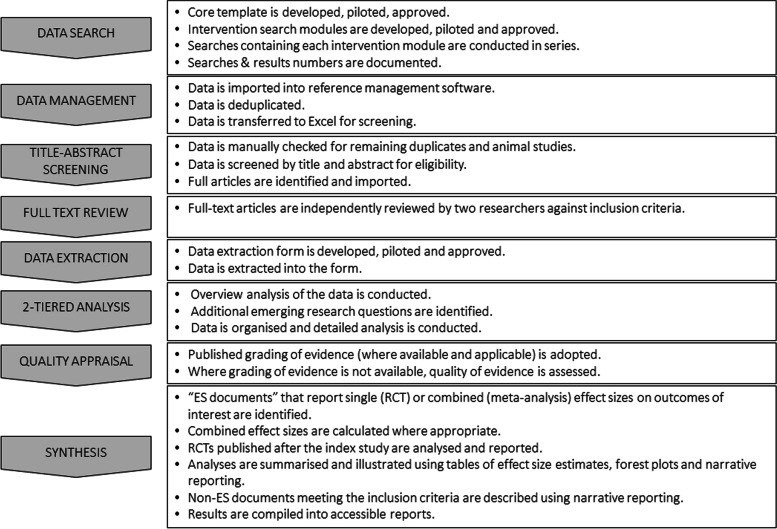
Overview and summary of the key stages of the Modular Review method

We developed a literature search model using an adaptation of the PICOS framework where I (intervention) was each of the 43 interventions stemming from the expert workshop. For each search, Population, Outcome and Study design modules were the same. The population was pregnant women, the outcome was LBW and related outcomes of preterm birth (PTB), small-for-gestational age (SGA) and stillbirth (SB), and the study design was controlled study designs (original reports, systematic reviews, meta-analyses, and reviews of reviews of RCTs). Comparison was omitted as it was not possible to define the broad range of relevant control types, for example routine care, different types of counselling, placebo products and alternative nutritional products. The Intervention module was different for each search (Fig. 1). Figure 2 illustrates the key steps of the review process.

We proceeded to convert the Population, Outcome and Study design parts of the model into a search strategy template for MEDLINE (Additional file 2). The template was constructed in Microsoft Word. Each element consisted of subject terms (Medical Subject Headings MeSH) and free text terms for title, abstract and keyword heading fields. The MEDLINE template was piloted and adjusted to address issues as they arose. The search strategy development involved identifying terms through database specific thesauri and from known relevant studies as well as utilising the collective expertise of the research group. We did not use any specific search filters although selected elements from several filters were included. The strategies and included terms were iteratively developed and tested for performance, until consensus was reached. While there was no formal peer review of search strategies using tools, such as the PRESS checklist, the two information specialists in the core research group were involved in developing each search strategy, providing an element of peer appraisal to each other’s work. A subset of strategies was reviewed by a third information specialist.

Once approved by consensus, the template was “translated” manually (i.e. without the use of automatisation tools) into equivalent searches for the other databases. The template was prepared for the following databases: MEDLINE (OvidSP), Embase (OvidSP), Cochrane Database of Systematic Reviews (Wiley Cochrane Library), Cochrane Central Register of Controlled Trials (Wiley Cochrane Library), CINAHL Complete (EbscoHOST).

We then designed the search strategy for the intervention modules following the same method described above. Search terms were related to either known risk factors for LBW (e.g. “bacterial vaginosis” or “smoking”) or alternatively, terms related to an intervention (e.g. “lipid-based nutrient supplements”) (Additional file 1). This broad approach, which we borrowed from the tradition of mapping studies, enabled us to

- Map all possible interventions, including new and emerging (e.g. the search term “depression” finds everything from yoga to antidepressants if they have been researched with controlled methods with respect to LBW).
- Identify risk factors that we may have not considered with respect to LBW (e.g. the search term “vaccination” found infection risks not considered in the workshop)

Out of the 43 research questions, some questions that would share similar search terms, were merged into one search. For instance, the investigation of the impact of conditional or unconditional cash transfer to pregnant women on birth outcomes was conducted within one search.

### Literature search

Searches were conducted during a four-month period, from March to June, 2020. In total, we conducted 33 literature searches in each of the five databases (see Additional file 3 for an exemplar search strategy). None of the searches were limited by year of publication or language.

The retrieved records were downloaded into RefWorks bibliographic management software for deduplication. Each specific search question was assigned a folder in RefWorks, and each folder had subfolders for individual databases. For each record, we added the database name, using RefWorks’ Global Edit function. References were imported into database subfolders as well as into the main folders. Deduplication was performed in the main folder of each research question.

### Study selection

Citations, including titles and abstracts (approximately 35,000), were entered into a Microsoft Excel-based repository on a shared work platform (Microsoft OneDrive). Titles and abstracts of the retrieved records were screened for eligibility by six research assistants. Our shared repository included functions to record several data elements such as the decision on eligibility, the rationale for the decision and who has made the decision. Due to the large number of records and time constraints, this screening step was undertaken in a single reviewer manner with the following quality control methods. Firstly, extensive training was given throughout the screening phase. This consisted of practice, feedback and regular meetings. Secondly, regular checks were made by a senior researcher (AK). Finally, occasional second reviews were undertaken in which the same randomly selected data was dual reviewed by two research assistants and possible discrepancies were discussed and solved in training meetings.

The inclusion criteria were derived from the PICOS framework as follows

- Relevant in terms of population
- Relevant in terms of intervention
- Reports at least one outcome of interest as primary or secondary outcome in a usable format
- Relevant in terms of research design
- Full article available in English

Articles were excluded if they did not meet the inclusion criteria. The same inclusion and exclusion criteria were broadly applied to all interventions. However, some interventions required additional specifications to the inclusion/exclusion criteria. For example, studies of interventions, such as cervical cerclage, that are known to have different outcomes in twin pregnancies had twin and multiple pregnancies as an additional exclusion criterion. At the title-abstract screening stage, the research assistants were advised to err on the side of caution and include records where some doubts existed regarding eligibility, particularly regarding outcomes as this information may be unclear in abstracts.

A second Microsoft Excel-based repository was set up for the records that were deemed eligible at the title-abstract screening stage. Full text articles (approximately 6000) were uploaded into separate files on Microsoft OneDrive for each intervention. Full texts were independently reviewed by two researchers for relevance against the inclusion/exclusion criteria. We also included records of relevant RCTs in the Cochrane Central Register of Controlled Trials. Studies that had started before 2010 but for which no publication of results could be found were presumed to be discontinued and were excluded. The post-2010 RCT records were presumed to be in progress. These records represented novel, emerging interventions for which there may not yet be an established evidence base.

The inclusion choices by the two independent reviewers were made visible in review meetings with at least three members of the team present and disagreements were discussed until consensus was reached. Reference lists of eligible articles were also checked for additional relevant studies.

### Data extraction

Data was extracted from the 365 selected articles in a manner that provided the foundation for analysing, summarising and interpreting the body of evidence. An Excel-based form was developed and piloted using a sample of selected studies. We systematically extracted data on study design, participants, intervention, comparison and outcome characteristics as well as geographical context. This was done as a single-extraction with the following quality control measures. Firstly, there was a division of labour between the extraction of basic information and estimates of effect size for each record, with the former undertaken by research assistants and the latter undertaken by members of the core research team (AK, PH, PNG, YM). Secondly, random checks of approximately 20% of the data for each intervention were undertaken by a member of the core research team. Automated sorting of the data was used to ensure that the population of the forms was complete and accurate.

### Analysis

When defining the search strategies, we deliberately kept the Intervention modules relatively broad by searching either the risk factor or the intervention. This resulted in a relatively large and transdisciplinary dataset for each research question. We proceeded with a two-tiered analysis. In the first round, we formed an *overview* of all data reporting a potentially large and diverse body of interventions and risk factors. We considered each of our 43 research questions and additionally identified novel, interesting questions emerging from the data. We then decided on the final, specific 46 research questions that would be answered with this data. The second tier of analysis consisted of a systematic *detailed analysis* relating to each specific research question. Part of this analysis was to identify studies that could be pooled and subjected to a meta-analysis.

### Appraisal

The ability to provide decision-makers with reliable information for priority setting requires that the evidence meets an adequate standard of quality. Systematic reviews often use Grading of Recommendations Assessment, Development, and Evaluation (GRADE) system for rating the quality of evidence [20]. Within our data, quality and, in some cases, certainty was often already assessed in a transparent fashion by the authors of systematic reviews. Thus, we developed a customised system of quality assessment that relied on the grade given in a systematic review if available and applicable. If a quality assessment was not available or applicable, we assessed the quality ourselves by assessing the risk of bias within individual studies (selection bias, performance bias, detection bias, attrition bias, reporting bias and any other bias) and within the body of evidence (publication bias) applying the GRADE approach as set forth in Cochrane Handbook [21].

### Synthesis

In order to synthesise the body of evidence into estimates of effects on the outcomes, we sought to identify reliable, comprehensive and recent sources of data. These source documents for effect size (ES) estimates, or “ES documents”, were identified through a hierarchical ordering of the various types of evidence according to Table 1. We sought the most recent examples of the highest level of evidence available for the effect size of each intervention.

**Table 1:** Hierarchy of evidence

Level	ES document (used for effect size estimation)	Included studies
1	A review of reviews of RCTs	Overview; umbrella review; meta-review; (systematic) review of (systematic) reviews
2	A systematic review of RCTs from Cochrane collaboration	Systematic review, meta-analysis and their combination
3	Other systematic review of RCTs	Systematic review, meta-analysis and their combination
4	RCTs in which case they were considered equally relevant ES documents from which we calculated the combined effect size	RCTs
5	Non-randomised controlled studies in cases were true randomisation was not feasible or ethical	Non-randomised controlled studies

Systematic reviews or reviews of reviews are not always up-to-date and the median update time for a systematic review is more than 5 years [22]. Therefore, in addition to choosing the latest reviews as ES documents, we also included RCTs published after the review. In all reporting of effect size, we used relative risk or odds ratio with 95% confidence interval, stating the number of randomised participants. The relative risk from a review and the relative risk from RCTs published after were reported separately.

To complete the synthesis, we combined the estimate of effect size (where available), the appraisal of quality and the overall availability of evidence for each intervention into a single classification system to enable cross-comparison. Colour codes and standardised statements were used to indicate the classification (Table 2).

**Table 2:** Classification of the evidence

Colour code	Standardized statement	Situations included
1. White	Unknown effect: Insufficient published research on the intervention’s effect on the outcome.	No RCTs, one low quality RCT with any result, or
		One moderate-to-high quality RCT where 95% CI of the RR includes 1, or
		Only narrative reporting
2. Grey	Unknown effect: Inconclusive published research on the intervention’s effect on the outcome.	At least two RCTs, 95% CI of the point estimate for a relative risk crosses widely on both sides of 1 (ranges from < 0.5 to > 2)
3. Green	Positive effect: The intervention likely reduces the risk of the adverse outcome.	At least two moderate-to-high quality RCTs included in a meta-analysis or IPD analysis, 95% CI of the point estimate of the RR is entirely below 1
4. Yellow	Possible positive effect: The intervention may reduce the risk of the adverse outcome.	At least two RCTs included in a meta-analysis or IPD analysis, 95% CI of the point estimate of the RR is entirely below 1, but there is concern about the quality of the data, or
		at least two moderate-to-high quality RCTs included in a meta-analysis or IPD analysis, 95% CI of the point estimate of the RR includes 1 but 90% CI of the point estimate of the RR is entirely below 1, or
		One moderate-to-high quality RCT, 95% CI of the point estimate of the RR is entirely below 1
5. Red	No positive effect: The intervention is not likely to reduce the risk of the adverse outcome.	Other situations, including meta-analysis results suggestive of harm

We documented the results for each intervention in a standardized, user-friendly report format (Additional file 4) along with a summary report that tabulated the results for all interventions. In order to enhance the applicability of the review findings, we listed the country or countries where the RCTs were conducted. For interventions where there was limited data from RCTs or only emerging evidence, we also presented non-randomised controlled studies and/or records from on-going studies, such as RCT register records. The reports and summary were submitted for review by the international expert panel of 51 members.

### Assessment of the Modular Review method

We conducted an assessment of the searching and screening methods, as well as an audit of the time spent from the inception of the project to when the results were available for use. We took measures before and during the search stage to ensure that our searches were capable of finding as many relevant records as possible and assessed how searches performed in this respect. These measures included that the search phase was led by information specialists in order to ensure compliance with principles of best practice. In the pilot phase, we drew on our own expertise and that of the wider global health community to identify a pre-specified set of articles that we would expect the search to find. We also reviewed irrelevant records brought in by the search and made adjustments where appropriate.

We assessed the possibility that our search may have missed relevant articles using the following tests. First, we selected 29 research questions for which we conducted additional free-text searches in other databases and data sources. We searched Google Scholar, Scopus, Web of Science, Science Direct, JSTOR and Academic Search Complete. Results were sorted by relevance and the first ten pages or 100 results were screened for relevant records. These 29 questions were chosen on the basis that the search was estimated to have a hypothetical risk of missing relevant records due to the paucity of established terminology in describing the risk or intervention.

Secondly, the intervention reports and summary were reviewed by the international expert panel of 51 members. They met in small expertise-specific groups to discuss the results and provide feedback, including whether they could identify articles that the review might have missed.

Additionally, we calculated the precision rate of the searches using the total number of selected records/the total machine deduplicated records.

Finally, we conducted an audit of the hours spent working on the project according to the category of task. All staff, student and contract researcher hours were included in the audit.

## Results

### Summary of results

**Fig. 3:**
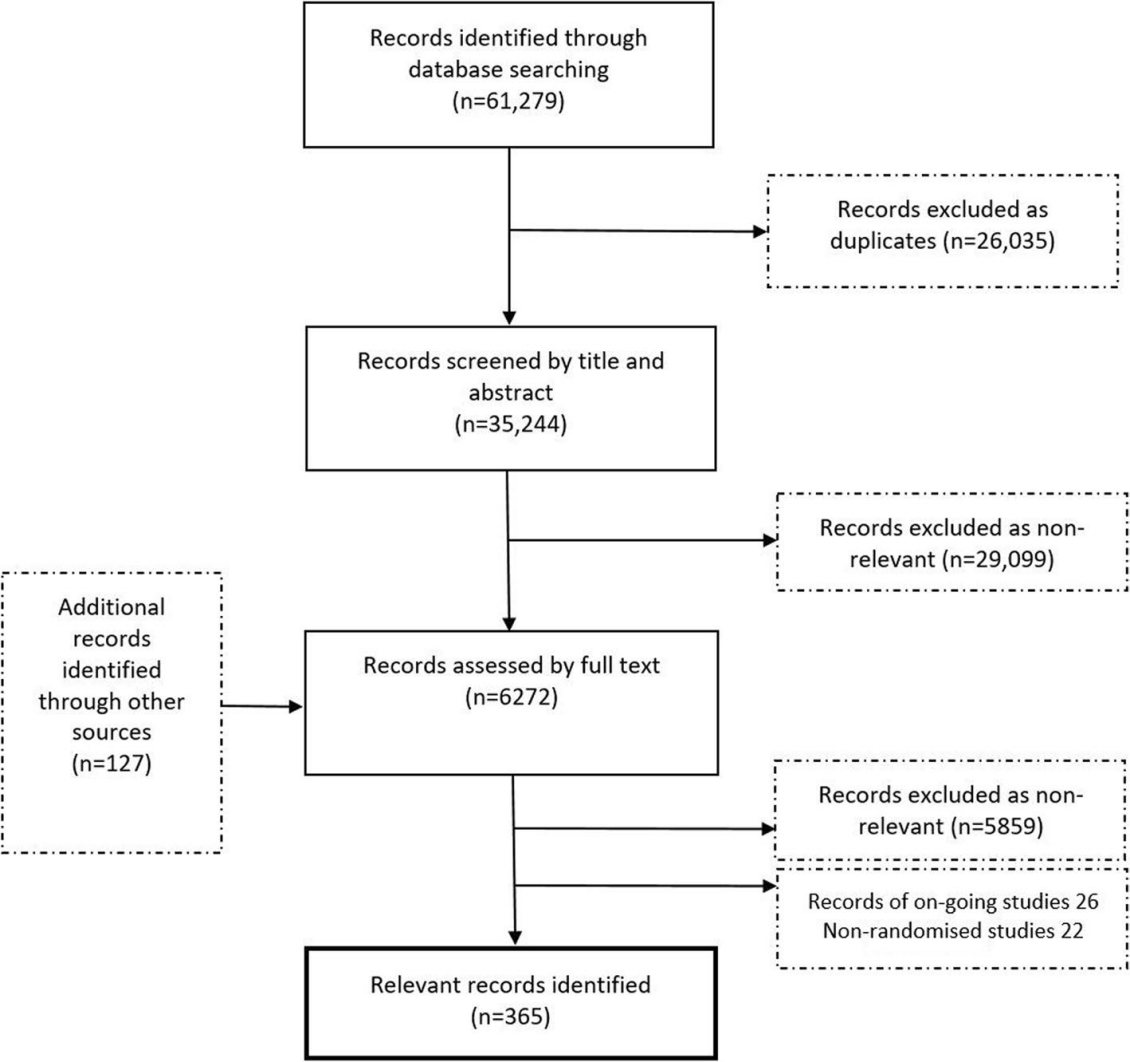
PRISMA 2009 flow chart of literature search and screening [23]

Our searches found 61,279 records (Fig. 3). After removal of duplicate records, we had 35,244 records to screen by abstract. Two researchers independently reviewed 6272 full text articles for relevance against inclusion/exclusion criteria. We included 365 relevant articles based on completed RCTs to provide 46 syntheses. In these syntheses, we addressed not only the questions set at the inception workshop, but additional questions that arose as a result of broad searching using either the risk factor or the intervention. For example, the search for vaccination (immunisation, inoculation) had the potential to capture RCTs of effects on LBW for vaccinations against viral influenza, *Haemophilus influenzae* B (HiB) and tetanus, all of which were synthesised as separate questions. Of these, tetanus was not in the original list of risk factors and would have therefore been missed if the search had risk-specific search terms. Thus, when the risk or the intervention was clearly definable with established terminology, the search was equally capable of finding unexpected risks or interventions as finding those that were expected. Moreover, we provided data on 26 ongoing trials and 22 non-randomised studies for 20 of the 46 research questions where the evidence base was limited or less established. In addition, we produced a list of approximately 15 interventions of potential interest for future research.

### Results of the assessment of the Modular Review method

The 29 additional free-text searches that were conducted to assess the possibility that the search and screening procedures might have missed relevant records produced 25 records. On closer inspection, 15 did not meet the inclusion criteria, two were already in the dataset and four were in reference lists of identified eligible articles. The remaining four records were likely to have been missed without this test. One was mistakenly screened as ineligible in the title/abstract screen, one failed to report the use of a control group, one was a record of an on-going study with no apparent reason for the search to miss it, and one failed to report outcomes of interest in the abstract although they were in the full article. In their review of the reports, the members of the international expert panel were asked to comment on the coverage of the literature and if there were any articles that were missing. Most responded that there were no relevant records missing from the review. All studies suggested in response to this question were found to be included. Taken together, the result of our assessment was that the coverage of the relevant literature by the review method was adequate. The overall search precision of the project was approximately 1% with variation across the searches attributed to availability of established terminology for specific interventions and risks.

### Resource use in the Modular Review method

The time from the inception of the project to when the results were available for use was 12 months. In total, the active time used in the review was 9360 working hours, i.e. approximately six full-time equivalent (FTE) years. Of this, approximately 60% was spent conducting the central research activity by the researchers in the core research group and 5% was expertise by information specialists (Table 3). One of the researchers in the core research group (AK) was employed in a second role as a project manager. Approximately 20% of her time was spent on training and supervising junior researchers (research assistants providing research support).

**Table 3:** Breakdown of work contributions

Contribution category	Working hours	Percentage
Senior leadership	190	2%
Research	5630	60%
Information specialist expertise	420	5%
Research support	2800	30%
Statistical support	320	3%
Total	9360	100%

## Discussion

This report describes a new method for gathering evidence in support of multifaceted, strategic initiatives to address major health challenges. The Modular Review method can be used to address complex research questions where there are multiple possible interventions that may be medicinal, surgical, nutritional, environmental and psychosocial. We utilised the PICOS framework to search for a variety of interventions, including the unanticipated and novel, by using a template where Population, Outcome and Study design modules are constant and the Intervention module varies with intervention or risk of interest. The end-result of the hierarchy-based synthesis is a list of potential interventions with estimates of effect size where this information is available. Where information on effectiveness is lacking, this method allows for narrative reporting and projection of future impact.

### Strengths and limitations of the Modular Review

#### Search

There are other established review methods in health research that could have been applied in this project including systematic reviews, scoping reviews, overview of systematic reviews and rapid reviews - all with their own strengths and limitations (Table 4). Systematic reviews are often considered to be the gold standard to search for, collate and synthesise the best available evidence [24]. The Modular Review employs a systematic PICOS-based approach known from systematic reviews but instead of one focused research question it addresses multiple, even dozens of research questions within the same review. The broad design of the intervention modules (specifying either the risk or the intervention) enabled the “discovery” of risk factors and interventions that were unknown at the inception workshop. It also facilitated the assessment of the potential nature, size and scope of the available evidence, which is typically the aim of scoping reviews. Use of a standard search template ensured consistency of focus across the project.

**Table 4:** Comparison of review types

	Systematic review *excluding qualitative systematic reviews	Scoping review	Overview of systematic reviews	Rapid review	Modular review
Description	Seeks to systematically search for, appraise and synthesise research evidence, adhering to guidelines on the conduct of a review [25]	Preliminary assessment of potential size, nature and scope of available research literature [12]	Bring together, appraise and synthesise evidence in areas where multiple systematic reviews already exist [26]	Search for and review evidence within limited timeframe and scope [27]	Combines systematic modular search process with stepwise synthesis and appraisal of the evidence to produce ‘best’ evidence synthesis. Focuses on broad condition or problem for which there are competing interventions
Expected timeframe	24 months or more [5]	0.5–20 months, mean length around 6 months [28]	6–18 months [3]	< 6 months [9]	6–18 months
Search	Comprehensive, systematic [25]	Broad [29]	Comprehensive, systematic [26]	Limited by time and resource constraints [27]	Comprehensive, systematic. Modular search allows searching on up to 50 modular variations.
Screening	Title-Abstract: Double independent screening recommended [30] Full text: Double independent screening [30, 31]	Title-Abstract: No established method but double independent screening recommended [14, 29] Full text: No established method but double independent screening recommended [29]	Title-Abstract: No established method but double independent screening recommended Full text: No established method but double independent screening recommended [3]	Title-Abstract: Often single screening, dual screen of at least 20% of abstracts recommended [9] Full text: Often single screening [9, 32]	Title-Abstract: Single screening with quality control measures Full text: Double independent screening
Data Extraction	Double independent extraction [33]	Double independent extraction recommended [14, 29]	Double independent extraction [34]	Single extraction, quality control measures recommended [9, 32]	Single extraction with quality control measures
Appraisal	Formal quality appraisal [35]	Formal quality appraisal often omitted [14, 36]	Formal quality assessment of included SRs; risk of bias of primary studies can be reported or independently assessed [37]	Formal quality appraisal often omitted [32] but recommended [9]	Relies primarily on existing quality assessment, quality assessment gaps filled as needed.
Synthesis	Uniform narrative and tabular synthesis on all data, often with meta-analysis [38]	Narrative and tabular without meta-analysis [14, 39]	Narrative and tabular synthesis, usually from systematic reviews [37]	Narrative and tabular [40]	Narrative and tabular synthesis on all data. Meta-analysis of data from “ES documents” where appropriate.

Other review types, such as rapid reviews, that aim for an adequate level of comprehensiveness whilst simultaneously working under time pressure [9, 41], employ “shortcuts” particularly in the search phase to manage the number of retrieved records. Some of the common shortcuts are (i) narrowing search criteria, (ii) applying date restrictions, (iii) limiting number of databases (iv) omitting the iterative process of searching and search term selection (v) leaving out expert consultation (i.e., librarian or information specialist for search strategy development) [42, 43]. We eschewed these shortcuts to avoid the exclusion of relevant studies and the introduction of selection bias [43]. However, we narrowed the final inclusion criteria to the English language. Whilst we accept that the exclusion of non-English articles limits the comprehensiveness of the results, it was not feasible to select or translate these articles within the given time constraints. The international expert panel did not identify any missed non-English language articles suggesting that a reasonable balance of trade-offs was achieved.

Unlike standard practice with scoping reviews [44], two information specialists were involved in the search strategy development and provided internal peer review as part of each strategy’s approval. The uptake of using of peer review in search strategy development has been low [45]. This may account for the high number of errors in search strategies, even in Cochrane reviews [46]. In light of this, we acknowledge that although our search strategy development work was thorough, a more formal audit of its components might have been beneficial.

#### Screening and data extraction

The number of records retrieved in the search was large due to the number of research questions, the adoption of a comprehensive approach without major shortcuts and the broad design of the searches, many of which specified only the risk factors. The management of such large datasets required special attention. Conducting the title-abstract screening with a single reviewer to remove irrelevant records saved time and resources. We recognize that double-screening is generally recommended [31]. It is possible that some relevant articles were missed due to single reviewer screening and one such case was revealed in our assessment. However, we employed training of reviewers, constant feedback, opportunities to ask questions, spot checks and double-screening of a proportion of the search results, all of which enhanced the fidelity of the process. Similarly, data extraction was managed by senior researchers as a single extraction process with quality control measures that included spot checks and the occasional dual extraction. These steps were taken to overcome the limitations of the single extraction approach.

#### Appraisal

A formal quality appraisal is a key part of the systematic review process whereas scoping reviews and rapid reviews tend to omit this step [32, 36]. The Modular Review method takes the middle road by only assessing the quality of the RCTs identified as ES documents and relying on the transparent appraisals that are already available in Cochrane and other high-quality systematic reviews. Whilst this approach saves time and resources, it may introduce inconsistencies through variation in appraisal styles among systematic reviews, and also between the studies assessed by our working group and other researchers. To avoid these pitfalls, we systematised and documented our appraisal process so that it was transparent and reproducible and all results could be traced back to decision points if needed.

#### Analysis and synthesis

Systematic reviews typically synthesise all original studies that meet their criteria, often with a meta-analysis. For some of our research questions, there was already an established evidence base with a recent systematic review providing a meta-analysis, thus obviating the need for this undertaking. We could have conducted an overview of reviews however this would have presented other challenges. First, there were research questions with limited data and no reviews available. Second, conducting a meta-analysis of the included reviews in an overview of reviews is challenging if the included reviews themselves already contain meta-analyses. Data from individual studies should not be used more than once as this may result in a misleading, and possibly overly precise estimate [3]. The solution to this issue would be to dismantle each review and recombine the results of the individual, included studies, which is complex and time-consuming [3]. We overcame this challenge by reporting on source documents for effect size (ES documents) based on the hierarchy of evidence. The reviews and studies published earlier than the ES documents were not included in the estimate of the effect, but were reported narratively, highlighting the possible overlap with the ES documents, as well as whether the results were deviating from or confirming the results of the ES documents.

#### Time constraints

Systematic reviews can be resource-intensive and may take several years [5, 47]. This is problematic for various groups of decision-makers in health, such as the developers of policy and clinical guidelines, as they need to make recommendations within limited timelines [41, 48]. Within the context of almost 50 research questions, adhering to strict systematic review guidelines would have been a barrier to timely results. Another option for us would have been to conduct a series of rapid reviews. The duration of these is typically considered to vary from 1 to 6 months [27]. However, a methodological exploration of 49 rapid reviews showed that the majority of the reviews were estimated to have taken 7–12 months from the completion of searching to publication [40]. Furthermore, the closer a standardised method is adhered to, the longer the review took to complete [40]. Hence, rapid review methodology does not necessarily provide a faster route to results and if it does, the number of short-cuts used is likely to be higher. Our approach of combining the principles of the systematic review methods in the search stage with streamlined analysis and synthesis enabled us to arrive at reliable conclusions in a timely fashion.

While we were able to work within a tight timeframe, the tradeoff in terms of resources was the employment and training of a larger team on short term contracts. Future versions of the Modular Review method may benefit from the automation of some of more high-throughput segments of the method. Steps such as the translation of searches to function in different databases may be automated by programs such as the Polyglot Search Translator [49]. Text mining using natural language processing could potentially save time and resources in the title-abstract screening step [50]. The importance of considering and reporting on the use of automation tools is underscored by their inclusion in the latest version (PRISMA 2020) of the PRISMA flow chart [51]. Further research is needed to understand the risks and benefits of automation in achieving and maintaining quality and fidelity in the review process.

### Implications for research and policy

The Modular Review can be used to inform research agendas. The comprehensive and comparative structure of the review provides a multi-disciplinary landscape view identifying gaps and caveats. It reveals needs for updates of systematic reviews and overarching umbrella reviews. It has the capability to identify emerging trends, overlaps and potential synergies. The search results form a dataset that may be developed into a more technologically advanced format, redeployable for further analyses.

This new review method is designed to support high-level decisions and policy making by providing evidence on a range of both practised and potential interventions spanning different disciplines and geographical contexts. Because the evidence has been gathered using the same search strategy, there is no need to try to reconcile differences in focus across a collection of individual reviews. The accessible output is ready for the next level of analysis such as cost-effectiveness and implementation planning. The robust but streamlined process enables the production of timely and reliable evidence. It facilitates comparisons, generalisations, and consolidation of strategic options, including holistic programmes to affect improvement in global health.

## Conclusion

We have devised the Modular Review, a novel systematic search and review method that is capable of providing and organising evidence on a broad range of interventions to tackle a health problem of global importance. Like the systematic review, this method aims to comprehensively search existing records for RCT and reviews of RCT and to gather and synthesize data toward the estimation of an intervention effect size. The modular approach enables the simultaneous synthesis of a diverse collection of interventions. The result is an accessible, reliable answer to the question of “what works?” Thus, it is an ideal method to support prioritisation, decisions and planning to implement an agenda for global health improvement.

## Supplementary Information

**Additional file 1.**

**Additional file 2.**

**Additional file 3.**

**Additional file 4.**

## Data Availability

The datasets used and/or analysed during the current study are available from the corresponding author on reasonable request.

## Abbreviations

ES: Effect size
FGR: Fetal growth restriction
IPD: Individual patient data
LBW: Low birth weight
LMIC: Low and middle income countries
PTB: Preterm birth
RCT: Randomised controlled trial
SB: Stillbirth
SGA: Small-for-gestational-age
SR: Systematic review
