# Supplementary material for: Modular literature review: a novel systematic search and review method to support priority setting in health policy and practice
Source: BMC Med Res Methodol. 2021 Nov 27;21:268. doi: 10.1186/s12874-021-01463-y (PMC8627616; doi:10.1186/s12874-021-01463-y)
Supplement: Supplementary file 1 — Additional file 1. [file 12874_2021_1463_MOESM1_ESM.docx]

List of potential interventions to reduce low birth weight

|  | Intervention | Adressed risk factors / conditions | Search terms |
| --- | --- | --- | --- |
| 1 | Vaccination | Bacterial and viral influenza, rubella, pertussis, tetanus in both mother and newborn. | vaccination, immunization, innoculation |
| 2 | Non-medical infection prevention (bednets, soap and WASH, reducing overcrowding, dietary restriction, advice on exposure avoidance) | Malaria, environmental enteric dysfunction, dysentry, helminth infection | Insecticide-treated bednet, indoor residual spraying, vector control, hand hygiene, soap, WASH, sanitation, toilet |
| 3 | Intermittent preventive treatment (IPT) | Malaria, placental malaria, anaemia | Intermittent preventive treatment in pregnancy (IPTp) |
| 4 | Treatment of worm infection | Worms, helminths, geohelminths, schistosomiasis | helminth, geohelminth, schistosomiasis, pinworm, threadworm, strongyloides |
| 5 | Treatment with antibacterial antibiotics | Bacterial vaginosis | bacterial vaginosis, anaerobic bacteria, Gardnerella, Atopobium, Ureaplasma, Mycoplasma, candidiasis |
| 6 | TORCH infection screening and treatment | TORCH infections | Toxoplasma, Rubella, CMV, Hepatitis, Zika, West Nile, Yellow Fever, LCMV, Listeria, Brucellosis, Dengue, Chikungunya, Herpes, Erythema Infectiosum, Varicella Zoster |
| 7 | Treatment of identified infections | Sexually transmitted infections | Chlamydia trachomatis, Neisseria gonorrhoea, Syphillis, HIV, Trichomonas, papilloma virus, group B Streptococcus |
| 8 | Treatment of oral/dental diseases | Periodontal disease, gingivitis, caries | periodontal disease, gingivitis, periodontosis, caries |
| 9 | Screening and treatment of urinary tract infections | Urinary tract infections | urinary tract infections, bacteriuria |
| 10 | Screening and treatment of tuberculosis | Tuberculosis (TB) | Tuberculosis |
| 11 | Dietary education to improve nutrient intake | Dietary inadequacy | Counselling search terms^1^, diet, body mass index, arm circumference |
| 12 | Micronutrient supplementation, single micronutirent | Micronutrient deficiency | Micronutrients, minerals, trace elements, zinc, selenium, copper, calcium, magnesium, chromium, sodium, potassium, manganese, iodine, fluorides, phosphates, vitamin A, vitamin B, vitamin C, vitamin D, vitamin E, vitamin K, iron, folate |
| 13 | Micronutrient supplementation, two or three micronutrients |  |  |
| 14 | Micronutrient supplementation, multiple micronutrients |  |  |
| 15 | Blanket balanced protein and energy supplementation | Undernourishment | Dietary protein, energy intake, amino acids, protein deficiency, malnutrition, fortified food, energy supplements |
| 16 | Screening and balanced protein and energy supplementation to undernourished women |  |  |
| 17 | Blanket lipid-based nutrient supplementation (LNS) | Dietary inadequacy | Lipid-based nutrient supplements, small quantity of energy (SQ-LNS), medium quantity (MQ-LNS), high quantity (HQ-LNS), nut, seed, pulse |
| 18 | Screening and targetted lipid-based nutrient supplements |  |  |
| 19 | PUFA and other fatty acids | Dietary inadequacy | Omega-3 polyunsaturated fatty acid (n-3 PUFA), docosahexaenoic acid, eicosapentaenoic acid, essential fatty acids, omega-6 PUFA, linoleic acid, linolenic acid, arachidonic acid, fish oil |
| 20 | Conditional cash transfer to all pregnant women | Poverty, lack of agency | Token economy, cash transfer, money, voucher, gift card, coupon, monetary incentive, financial incentive, demand-side financing, public assistance, financial support, social security, benefit, welfare, payment, transfer, aid, subsidize, allowance. |
| 21 | Unconditional cash transfer to all pregnant women | Poverty, lack of agency |  |
| 22 | Monitoring and promoting maternal gestational weight gain | Insufficient maternal weight gain | weight gain |
| 23 | Screening and tailored antenatal follow-up scheme for women with previous miscarriage, stillbirth, LBW or preterm baby | Repeat miscarriage, stillbirth, LBW or preterm baby | cervical length measurement, trans vaginal ultrasound, increased antenatal follow-up, high-risk pregnancy |
| 24 | Screening and tailored antenatal follow-up scheme for women with low age or primiparity | Low maternal age, primiparity | teenager, first time mother, low gynaecological age, primiparous, nulliparous |
| 25 | Intensified fetal growth monitoring | Unidentified fetal distress | enhanced fetal growth monitoring, intrauterine growth retardation, in utero growth restriction |
| 26 | Screening for pre-eclampsia with blood pressure measurement and urine dipstick and referral of women with pre-eclampsia for secondary or tertiary care | Pre-eclampsia, placental dysfunction | Pre-eclampsia, toxemia, pregnancy-induced hypertension, placental dysfunction |
| 27 | Low-dose aspirin for women at risk of pre-eclampsia or pregnancy-induced hypertension |  |  |
| 28 | Calcium supplementation (low-dose or high dose) for women at risk of pre-eclampsia or pregnancy-induced hypertension |  |  |
| 29 | Screening and targeted support to women experiencing domestic violence | Physical and emotional, acute or chronic harm, distress, stress, trauma, depression, mental health problems | Physical, domestic, interpersonal, spousal abuse, intimate partner violence, gender-based violence, assault, battered women, stress, anxiety, psychiatric disorder, depression, mood, self-esteem, mental health, psychosocial wellbeing, emotional balance |
| 30 | Blanket support for mental health during pregnancy |  |  |
| 31 | Screening and targeted support for women with mental health problems |  |  |
| 32 | Screening and treatment for women with gestational diabetes | Gestational diabetes | gestational diabetes, impaired glucose tolerance, fasting glucose, oral glucose tolerance test |
| 33 | Screening and special support for pregnant women with sickle-cell disease | Sickle-cell disease | Sickle cell anaemia, crescent cell disease, drepanocytosis |
| 34 | Promotion of smoking cessation | Smoking during pregnancy | counselling search terms^1^, smoking, tobacco, cigarette, cigar, pipe, vaping, nicotine, electronic nicotine delivery systems, water pipe, stop, end, quit, abstain, avoid, abstinence, cessation, reduction, prevention |
| 35 | Promotion of alcohol avoidance | Alcohol consumption during pregnancy | counselling search terms^1^, stop, end, quit, abstain, avoid, withdrawal, abstinence, alcohol, ethanol, drinking, unit |
| 36 | Reduction of household fuel pollution and ambient particulate matter exposure of pregnant women | Indoor or outdoor inhaled particulate matter exposure | Air pollution, particulate matter, carbon monoxide, nitrogen dioxide, indoor, ambient, household, solid fuel, cooking fuel, fire, stove, PM2.5, PM10, secondhand smoke, sulfur dioxide, domestic pollution exposure, solid biomass fuel, gas, coal, wood, crop residue, animal dung, traditional stove, ancient stove, chimney, flute, ventilation, diesel, kerosene, gasoline, toxic, emission, exhaust |
| 37 | Antenatal counselling about avoidance of aflatoxins or heavy metals | Aflatoxin and heavy metal exposure | Counselling search terms^1^, heavy metals, beryllium, copper, arsenic, zinc, selenium, molybdenum, cadmium, tin, antimony, lead, chromium, nickel, vanadium, mercury, lithium, yttrium, indium aflatoxin, mycotoxin, fungal toxin, Aspergillus, occupational, environmental exposure |
| 38 | Treatment of maternal environmental enteric dysfunction (EED) | Maternal EED, impaired nutrient uptake | Environmental enteric dysfunction, enteritis, intestinal disease, tropical sprue, enteric disease, enteropathy, malabsorption, inflammation |
| 39 | Progesterone therapy for women at risk of preterm birth | Maternal hormononal insufficiency | progesterone, 17-hydroxyprogesterone caproate, cyclogest |
| 40 | Uterine cervical cerclage | Uterine cervix insufficiency | Cervical incompetence, insufficiency, weakness, cervical cerclage, cervical stitch, suture, cervical ligation, Shirodkar, McDonald, trans-abdominal, trans-vaginal |
| 41 | Bed rest, avoidance of physical exercise | Excessive physical exercise during pregnancy | exercise, activity, aerobic, resistance, physical, strength, endurance, training, low, avoid, lack, stop, reduce, decrease, restrict |
| 42 | Counselling of women living in high altitude about moving to lower altitude during pregnancy | Low oxygen pressure due to residence in high altitude | Altitude, highland, mountain, lowland |
| 43 | Antenatal counselling about modes of delivery | Preterm induction of labour or preterm caesarean section for non-medical reasons | Counselling search terms^1^, mode of delivery, delivery options, non-medical, non-indicated, unnecessary, induction of labour, planned Caesarean birth, elective, surgical, scheduled labour induction, c-section, vaginal birth, parturition |
|  |  | ^1^*Counselling search terms* | *Counselling, motivational interviewing, education, promotion, handout, prevention, advice, recommendation, guidance, teaching, programme, instruction* |
