# Supplementary material for: Modular literature review: a novel systematic search and review method to support priority setting in health policy and practice
Source: BMC Med Res Methodol. 2021 Nov 27;21:268. doi: 10.1186/s12874-021-01463-y (PMC8627616; doi:10.1186/s12874-021-01463-y)
Supplement: Supplementary file 2 — Additional file 2. [file 12874_2021_1463_MOESM2_ESM.docx]

| **Search strategy template for Medline (OVID)** Ovid MEDLINE(R) and Epub Ahead of Print, In-Process & Other Non-Indexed Citations and Daily | |
| --- | --- |
| 1 | pregnancy/ or Pregnant Women/ or Mothers/ or Maternal health/ |
| 2 | (pregnan* or trimester* or mother* or matern* or "expecting wom*" or "expecting mother*" or "expecting femal*" or "expectant wom*" or "expectant mother*" or "expectant female*" or antenatal* or prenatal* or pre-natal* or "pre natal*" or post-conception* or "post conception*" or postconception*).ti,ab,kf. |
| 3 | (gestat* and (women* or woman* or female* or mother* or matern*)).ti,ab,kf,sh,kw. |
| 4 | or/1-3 |
| 5 | Intervention-specific subject heading terms |
| 6 | Intervention-specific free text terms (ti,ab,kf) |
| 7 | or/5-6 |
| 8 | exp infant, low birth weight/ or Birth Weight/ or Fetal Weight/ or Infant, Small for Gestational Age/ |
| 9 | (lbw or vlbw or elbw or "birth weight*" or "weight at birth" or "neonatal weight*" or "neonatal measure*" or "neonatal length*" or "newborn weight*" or "newborn measure*" or "newborn length*" or "low weight*" or "small for gestational age" or "small-for-gestational age" or sga or "fetal weight*" or "foetal weight*" or "fetal growth" or "foetal growth" or "fetal measure*" or "foetal measure*" or "fetal length*" or "foetal length*" or "birth length*" or "birth anthropometr*" or "birth measure*" or "infant measure*" or "head circumferen*" or ("weight for age" adj3 birth) or (anthropometr* adj3 birth) or (small* adj3 (babies or neonat* or newborn*)) or (small adj3 "gestational age")).ti,ab,kf. |
| 10 | (iugr or fgr or ((fetal or foetal or "in utero" or intrauterine or intra-uterine) adj3 ("growth restrict*" or "growth retard*" or "growth disturb*" or "growth abnormalit*" or "growth disorder*" or "growth trajector*" or "length trajector*" or "weight trajector*"))).ti,ab,kf. |
| 11 | exp infant, premature/ or Premature Birth/ or Obstetric Labor, Premature/ or Infant, Extremely Premature/ |
| 12 | (prematurity or ((prematur* or preterm* or pre-term*) adj3 (babies or neonat* or newborn* or birth* or childbirth* or delivery or labor or labour or parturiti*))).ti,ab,kf. |
| 13 | Fetal Membranes, Premature Rupture/ or Uterine Cervical Incompetence/ |
| 14 | ("Premature ruptur*" or "Preterm ruptur*" or "Prelabor ruptur*" or "pre-labor ruptur*" or "Prelabour ruptur*" or "pre-labour ruptur*" or "pprom" or "cervical incompetence" or "cervical insufficiency" or "cervical weakness").ti,ab,kf. |
| 15 | Fetal Death/ or Fetal Mortality/ or Stillbirth/ |
| 16 | (stillbirth* or still-birth* or "still birth*" or stillborn* or still-born* or "still born*" or ((fetal or foetal or antepartum or intrapartum or antenatal* or prenatal* or intrauterin* or intra-uterin* or "in utero") adj3 (loss or death* or mortality or morbidity or demise))).ti,ab,kf. |
| 17 | ((neonatal or birth) adj3 outcome*).ti,ab,kf. |
| 18 | or/8-17 |
| 19 | exp clinical trial/ or clinical trials as topic/ or random allocation/ or exp randomized controlled trial/ or randomized controlled trial as topic/ or meta-analysis/ or Meta-Analysis as Topic/ or systematic review/ or control groups/ or double-blind method/ or single-blind method/ |
| 20 | (clinical trial or controlled clinical trial or randomized controlled trial or meta-analysis or systematic review).pt. |
| 21 | (rct or cct or "random allocat*" or randomly or randomized or randomised or cluster-random* or "cluster random*" or "stepped wedge" or "clinical trial" or "controlled study" or "controlled trial" or "control group*" or blinding or blinded or masking or "double-blind*" or "single-blind*" or "systematic review*" or meta-analysis or (systematic* adj2 (review* or overview*))).ti,ab,kf. or (trial or trials).ti. |
| 22 | or/19-21 |
| 23 | 4 and 7 and 18 and 22 |
| 24 | exp animals/ not humans.sh. |
| 25 | 23 and 24 |
| 26 | 23 not 25 |
