# Supplementary material for: Modular literature review: a novel systematic search and review method to support priority setting in health policy and practice
Source: BMC Med Res Methodol. 2021 Nov 27;21:268. doi: 10.1186/s12874-021-01463-y (PMC8627616; doi:10.1186/s12874-021-01463-y)
Supplement: Supplementary file 3 — Additional file 3. [file 12874_2021_1463_MOESM3_ESM.docx]

**Full search strategies for INF 1 Vaccination**

**1. Ovid MEDLINE(R) ALL**
Database coverage: 1946 to March 02, 202>
Interface/URL: OvidSP
Search date: 3.3.2020
Number of records retrieved: 325
Search strategy:

1 pregnancy/ or Pregnant Women/ or Mothers/ or Maternal health/ (894231)

2 (pregnan* or trimester* or mother* or matern* or "expecting wom*" or "expecting mother*" or "expecting femal*" or "expectant wom*" or "expectant mother*" or "expectant female*" or antenatal* or prenatal* or pre-natal* or "pre natal*" or post-conception* or "post conception*" or postconception*).ti,ab,kf. (839709)

3 (gestat* and (women* or woman* or female* or mother* or matern*)).ti,ab,kf,sh,kw. (203914)

4 or/1-3 (1227485)

5 immunotherapy/ or exp immunization/ or exp vaccination/ or exp Vaccines/ (350647)

6 (vaccin* or immunizat* or immunisat* or immunotherap* or inoculat*).ti,ab,kf. (535876)

7 or/5-6 (637923)

8 exp infant, low birth weight/ or Birth Weight/ or Fetal Weight/ or Infant, Small for Gestational Age/ (68330)

9 (lbw or vlbw or elbw or "birth weight*" or "weight at birth" or "neonatal weight*" or "neonatal measure*" or "neonatal length*" or "newborn weight*" or "newborn measure*" or "newborn length*" or "low weight*" or "small for gestational age" or "small-for-gestational age" or sga or "fetal weight*" or "foetal weight*" or "fetal growth" or "foetal growth" or "fetal measure*" or "foetal measure*" or "fetal length*" or "foetal length*" or "birth length*" or "birth anthropometr*" or "birth measure*" or "infant measure*" or "head circumferen*" or ("weight for age" adj3 birth) or (anthropometr* adj3 birth) or (small* adj3 (babies or neonat* or newborn*)) or (small adj3 "gestational age")).ti,ab,kf. (95007)

10 (iugr or fgr or ((fetal or foetal or "in utero" or intrauterine or intra-uterine) adj3 ("growth restrict*" or "growth retard*" or "growth disturb*" or "growth abnormalit*" or "growth disorder*" or "growth trajector*" or "length trajector*" or "weight trajector*"))).ti,ab,kf. (19817)

11 exp infant, premature/ or Premature Birth/ or Obstetric Labor, Premature/ or Infant, Extremely Premature/ (77214)

12 (prematurity or ((prematur* or preterm* or pre-term*) adj3 (babies or neonat* or newborn* or birth* or childbirth* or delivery or labor or labour or parturiti*))).ti,ab,kf. (77558)

13 Fetal Membranes, Premature Rupture/ or Uterine Cervical Incompetence/ (6401)

14 ("Premature ruptur*" or "Preterm ruptur*" or "Prelabor ruptur*" or "pre-labor ruptur*" or "Prelabour ruptur*" or "pre-labour ruptur*" or "pprom" or "cervical incompetence" or "cervical insufficiency" or "cervical weakness").ti,ab,kf. (7670)

15 Fetal Death/ or Fetal Mortality/ or Stillbirth/ (28647)

16 (stillbirth* or still-birth* or "still birth*" or stillborn* or still-born* or "still born*" or ((fetal or foetal or antepartum or intrapartum or antenatal* or prenatal* or intrauterin* or intra-uterin* or "in utero") adj3 (loss or death* or mortality or morbidity or demise))).ti,ab,kf. (36536)

17 ((neonatal or birth) adj3 outcome*).

ti,ab,kf. (19964)

18 or/8-17 (259226)

19 exp clinical trial/ or clinical trials as topic/ or random allocation/ or exp randomized controlled trial/ or randomized controlled trial as topic/ or meta-analysis/ or Meta-Analysis as Topic/ or "systematic review"/ or control groups/ or double-blind method/ or single-blind method/ (1258828)

20 (clinical trial or controlled clinical trial or randomized controlled trial or meta-analysis or systematic review).pt. (1012201)

21 (rct or cct or "random allocat*" or randomly or randomized or randomised or cluster-random* or "cluster random*" or "stepped wedge" or "clinical trial" or "controlled study" or "controlled trial" or "control group*" or blinding or blinded or masking or "double-blind*" or "single-blind*" or "systematic review*" or meta-analysis or (systematic* adj2 (review* or overview*))).ti,ab,kf. or (trial or trials).ti. (1634613)

22 or/19-21 (2156342)

23 4 and 7 and 18 and 22 (400)

24 exp animals/ not humans.sh. (4673912)

25 23 and 24 (75)

26 23 not 25 (325)

**2. Cochrane Central Register of Controlled Trials (CENTRAL)**
Database coverage: Issue 12 of 12, December 2019
Interface/URL: Wiley Cochrane Library
Search date: 10.3.2020
Number of records retrieved: 342
Search strategy:

#1 [mh ^pregnancy] or [mh ^"Pregnant Women"] or [mh ^Mothers] or [mh ^"Maternal health"](2,801)

#2 (pregnan* or trimester* or mother* or matern* or antenatal* or prenatal* or pre-natal* or "pre natal*" or post-conception* or "post conception*" or postconception*):ti,ab,kw (76,813)

#3 (expect* NEXT (mother* or woman* or women* or female*)):ti,ab,kw (826)

#4 (gestat* and (women* or woman* or female* or mother* or matern*)):ti,ab,kw (18,906)

#5 #1 OR #2 OR #3 OR #4 (80,532)

#6 [mh ^immunotherapy] or [mh immunization] or [mh vaccination] or [mh Vaccines] (14,361)

#7 (vaccin* or immunizat* or immunisat* or immunotherap* or inoculat*):ti,ab,kw (34,852)

#8 #6 OR #7 (34,874)

#9 [mh "infant, low birth weight"] or [mh ^"Birth Weight"] or [mh ^"Fetal Weight"] or [mh ^"Infant, Small for Gestational Age"] (3,513)

#10 (lbw or vlbw or elbw or "weight at birth" or "low weight" or "low weights" or "low weighing or "small for gestational age" or "small-for-gestational age" or sga):ti,ab,kw (3,345)

#11 ((fetal or foetal or birth or newborn or neonatal) NEXT (length* or growth* or weight* or measure* or anthropometr*)):ti,ab,kw (11,416)

#12 (infant* NEXT measure*):ti,ab,kw (61)

#13 (head NEXT circumference*):ti,ab,kw (1,294)

#14 ("weight for age" NEAR/3 birth):ti,ab,kw (30)

#15 (small* NEAR/3 (babies or neonat* or newborn*)):ti,ab,kw (232)

#16 (small NEAR/3 "gestational age"):ti,ab,kw (925)

#17 (iugr or fgr):ti,ab,kw (479)

#18 ((fetal or foetal or "in utero" or intrauterine or intra-uterine) NEAR/3 (growth or length or weight*) NEAR/3 (restrict* or retard* or disturb* or abnormalit* or disorder* or trajector*)):ti,ab,kw (1,262)

#19 [mh "infant, premature"] or [mh ^"Premature Birth"] or [mh ^"Obstetric Labor, Premature"] or [mh ^"Infant, Extremely Premature"] (5,578)

#20 ((prematur* or preterm* or pre-term*) NEAR/3 (babies or neonat* or newborn* or birth* or childbirth* or delivery or labor or labour or parturiti*)):ti,ab,kw (9,736)

#21 (prematurity):ti,ab,kw (5,390)

#22 [mh ^"Fetal Membranes, Premature Rupture"] or [mh ^"Uterine Cervical Incompetence"] (470)

#23 ("pprom" or "cervical incompetence" or "cervical insufficiency" or "cervical weakness"):ti,ab,kw (364)

#24 ((Premature or preterm or pre-term or prelabor or prelabour or pre-labor or pre-labour) NEXT (ruptur*)):ti,ab,kw (1,130)

#25 [mh ^"Fetal Death"] or [mh ^"Fetal Mortality"] or [mh ^Stillbirth] (338)

#26 ((stillbirth* or still NEXT birth* or stillborn* or still NEXT born*)):ti,ab,kw (1,016)

#27 ((fetal or foetal or antepartum or intrapartum or antenatal* or prenatal* or intrauterin* or intra-uterin* or "in utero") NEAR/3 (loss or death* or mortality or morbidity or demise)):ti,ab,kw (1,355)

#28 ((neonatal or birth) NEAR/3 (outcome*)):ti,ab,kw (5,133)

#29 #9 OR #10 OR #11 OR #12 OR #13 OR #14 OR #15 OR #16 OR #17 OR #18 OR #19 OR #20 OR #21 OR #22 OR #23 OR #24 OR #25 OR #26 OR #27 OR #28 (26,852)

#30 (#5 AND #8 AND #29) in Trials (342)

**3. Cochrane Database of Systematic Reviews (CDSR)**
Database coverage: Issue 12 of 12, December 2019
Interface/URL: Wiley Cochrane Library
Search date: 10.3.2020
Number of records retrieved: 14
Search strategy:

#1 [mh ^pregnancy] or [mh ^"Pregnant Women"] or [mh ^Mothers] or [mh ^"Maternal health”] (2,801)

#2 (pregnan* or trimester* or mother* or matern* or antenatal* or prenatal* or pre-natal* or "pre natal*" or post-conception* or "post conception*" or postconception*):ti,ab,kw (76,813)

#3 (expect* NEXT (mother* or woman* or women* or female*)):ti,ab,kw (826)

#4 (gestat* and (women* or woman* or female* or mother* or matern*)):ti,ab,kw
 (18,906)

#5 #1 OR #2 OR #3 OR #4 (80,532)

#6 [mh ^immunotherapy] or [mh immunization] or [mh vaccination] or [mh Vaccines] (14,361)

#7 (vaccin* or immunizat* or immunisat* or immunotherap* or inoculat*):ti,ab,kw (34,852)

#8 #6 OR #7 (34,874)

#9 [mh "infant, low birth weight"] or [mh ^"Birth Weight"] or [mh ^"Fetal Weight"] or [mh ^"Infant, Small for Gestational Age"] (3,513)

#10 (lbw or vlbw or elbw or "weight at birth" or "low weight" or "low weights" or "low weighing" or "small for gestational age" or "small-for-gestational age" or sga):ti,ab,kw (3,345)

#11 ((fetal or foetal or birth or newborn or neonatal) NEXT (length* or growth* or weight* or measure* or anthropometr*)):ti,ab,kw (11,416)

#12 (infant* NEXT measure*):ti,ab,kw (61)

#13 (head NEXT circumference*):ti,ab,kw (1294)

#14 ("weight for age" NEAR/3 birth):ti,ab,kw (30)

#15 (small* NEAR/3 (babies or neonat* or newborn*)):ti,ab,kw (232)

#16 (small NEAR/3 "gestational age"):ti,ab,kw (925)

#17 (iugr or fgr):ti,ab,kw (479)

#18 ((fetal or foetal or "in utero" or intrauterine or intra-uterine) NEAR/3 (growth or length or weight*) NEAR/3 (restrict* or retard* or disturb* or abnormalit* or disorder* or trajector*)):ti,ab,kw (1,262)

#19 [mh "infant, premature"] or [mh ^"Premature Birth"] or [mh ^"Obstetric Labor, Premature"] or [mh ^"Infant, Extremely Premature"] (5,578)

#20 (prematurity):ti,ab,kw (5,390)

#21 ((prematur* or preterm* or pre-term*) NEAR/3 (babies or neonat* or newborn* or birth* or childbirth* or delivery or labor or labour or parturiti*)):ti,ab,kw 9736

#22 [mh ^"Fetal Membranes, Premature Rupture"] or [mh ^"Uterine Cervical Incompetence"] (470)

#23 ("pprom" or "cervical incompetence" or "cervical insufficiency" or "cervical weakness"):ti,ab,kw (364)

#24 ((Premature or preterm or pre-term or prelabor or prelabour or pre-labor or pre-labour) NEXT (ruptur*)):ti,ab,kw (1,130)

#25 [mh ^"Fetal Death"] or [mh ^"Fetal Mortality"] or [mh ^Stillbirth] (338)

#26 (stillbirth* or still NEXT birth* or stillborn* or still NEXT born*):ti,ab,kw (1,016)

#27 ((fetal or foetal or antepartum or intrapartum or antenatal* or prenatal* or intrauterin* or intra-uterin* or "in utero") NEAR/3 (loss or death* or mortality or morbidity or demise)):ti,ab,kw (1,355)

#28 ((neonatal or birth) NEAR/3 (outcome*)):ti,ab,kw (5,133)

#29 #9 OR #10 OR #11 OR #12 OR #13 OR #14 OR #15 OR #16 OR #18 OR #19 OR #21 OR #22 OR #23 OR #24 OR #25 OR #26 OR #27 OR #28 (26,852)

#30 (#5 AND #8 AND #29) in Cochrane Reviews, Cochrane Protocols (14)

**4. CINAHL COMPLETE**
Database coverage: from inception to date
Interface/URL: EBSCOhost
Search date: 10.3.2020
Number of records retrieved: 102
Search strategy:

S1 MH("Pregnancy" OR "Expectant Mothers" or "Mothers") (204,729)

S2 TI((pregnan* or trimester* or mother* or matern* or "expecting wom*" or "expecting mother*" or "expecting femal*" or "expectant wom*" or "expectant mother*" or "expectant female*" or antenatal* or prenatal* or pre-natal* or "pre natal*" or post-conception* or "post conception*" or postconception*)) OR AB((pregnan* or trimester* or mother* or matern* or "expecting wom*" or "expecting mother*" or "expecting femal*" or "expectant wom*" or "expectant mother*" or "expectant female*" or antenatal* or prenatal* or pre-natal* or "pre natal*" or post-conception* or "post conception*" or postconception*)) OR SU((pregnan* or trimester* or mother* or matern* or "expecting wom*" or "expecting mother*" or "expecting femal*" or "expectant wom*" or "expectant mother*" or "expectant female*" or antenatal* or prenatal* or pre-natal* or "pre natal*" or post-conception* or "post conception*" or postconception*)) (308,667)

S3 TI(gestat* and (women* or woman* or female* or mother* or matern*)) OR AB(gestat* and (women* or woman* or female* or mother* or matern*)) OR SU(gestat* and (women* or woman* or female* or mother* or matern*)) (42,290)

S4 S1 OR S2 OR S3 (312,548)

S5 MH("Viral Vaccines+" OR "Vaccines, Combined+" OR "Bacterial Vaccines+" OR "Vaccines" OR "Immunization" OR "Immunization Programs" OR "Immunotherapy" OR "Vaccination Coverage") (60,476)

S6 TI ( (vaccin* or immunizat* or immunisat* or immunotherap* or inoculat*) ) OR AB ( (vaccin* or immunizat* or immunisat* or immunotherap* or inoculat*) ) (62,033)

S7 S5 OR S6 (82,764)

S8 MH("Birth Weight" OR "Infant, Low Birth Weight" OR "Infant, Very Low Birth Weight" OR "Infant, Small for Gestational Age" OR "Fetal Weight" OR "Head Circumference") (21,018)

S9 TI ( lbw or vlbw or elbw or "birth weight*" or "weight at birth" or "neonatal weight*" or "neonatal measure*" or "neonatal length*" or "newborn weight*" or "newborn measure*" or "newborn length*" or "low weight*" or "small for gestational age" or "small-for-gestational age" or sga or "fetal weight*" or "foetal weight*" or "fetal growth" or "foetal growth" or "fetal measure*" or "foetal measure*" or "fetal length*" or "foetal length*" or "birth length*" or "birth anthropometr*" or "birth measure*" or "infant measure*" or "head circumferen*") ) OR AB ( lbw or vlbw or elbw or "birth weight*" or "weight at birth" or "neonatal weight*" or "neonatal measure*" or "neonatal length*" or "newborn weight*" or "newborn measure*" or "newborn length*" or "low weight*" or "small for gestational age" or "small-for-gestational age" or sga or "fetal weight*" or "foetal weight*" or "fetal growth" or "foetal growth" or "fetal measure*" or "foetal measure*" or "fetal length*" or "foetal length*" or "birth length*" or "birth anthropometr*" or "birth measure*" or "infant measure*" or "head circumferen*") ) (36,302)

S10 TI ("weight for age" N3 birth) OR AB ("weight for age" N3 birth) (451)

S11 TI (anthropometr* N3 birth) OR AB (anthropometr* N3 birth) (218)

S12 TI ( small* N3 (babies or neonat* or newborn*) ) OR AB ( small* N3 (babies or neonat* or newborn*) ) (1,086)

S13 TI (small* N3 "gestational age") OR AB (small* N3 "gestational age") (3,877)

S14 TI (iugr or fgr ) OR AB(iugr or fgr) (1,584)

S15 TI ((fetal or foetal or "in utero" or intrauterine or intra-uterine) N3 ("growth restrict*" or "growth retard*" or "growth disturb*" or "growth abnormalit*" or "growth disorder*" or "growth trajector*" or "length trajector*" or "weight trajector*")) OR AB ((fetal or foetal or "in utero" or intrauterine or intra-uterine) N3 ("growth restrict*" or "growth retard*" or "growth disturb*" or "growth abnormalit*" or "growth disorder*" or "growth trajector*" or "length trajector*" or "weight trajector*")) (4,315)

S16 MH("Labor, Premature" OR "Childbirth, Premature" OR "Infant, Premature") (31,251)

S17 TI (prematurity) OR AB (prematurity) (5,232)

S18 TI ( (prematur* or preterm* or pre-term*) N3 (babies or neonat* or newborn* or birth* or childbirth* or delivery or labor or labour or parturiti*) ) OR AB ( (prematur* or preterm* or pre-term*) N3 (babies or neonat* or newborn* or birth* or childbirth* or delivery or labor or labour or parturiti*) ) (22,176)

S19 MH("Fetal Membranes, Premature Rupture" OR "Cervix Incompetence") (1,974)

S20 TI ( ("Premature ruptur*" or "Preterm ruptur*" or "Prelabor ruptur*" or "pre-labor ruptur*" or "Prelabour ruptur*" or "pre-labour ruptur*" or "pprom" or "cervical incompetence" or "cervical insufficiency" or "cervical weakness") ) OR AB ( ("Premature ruptur*" or "Preterm ruptur*" or "Prelabor ruptur*" or "pre-labor ruptur*" or "Prelabour ruptur*" or "pre-labour ruptur*" or "pprom" or "cervical incompetence" or "cervical insufficiency" or "cervical weakness") ) (2,164)

S21 TI (stillbirth* or still-birth* or "still birth*" or stillborn* or still-born* or "still born*" ) OR AB ( stillbirth* or still-birth* or "still birth*" or stillborn* or still-born* or "still born*" ) (4,297)

S22 TI ( (fetal or foetal or antepartum or intrapartum or antenatal* or prenatal* or intrauterin* or intra-uterin* or "in utero") N3 (loss or death* or mortality or morbidity or demise) ) OR AB ( (fetal or foetal or antepartum or intrapartum or antenatal* or prenatal* or intrauterin* or intra-uterin* or "in utero") N3 (loss or death* or mortality or morbidity or demise) ) (4712)

S23 TI((neonatal or birth) N3 outcome*) OR AB((neonatal or birth) N3 outcome*) (10,744)

S24 S8 OR S9 OR S10 OR S11 OR S12 OR S13 OR S14 OR S15 OR S16 OR S17 OR S18 OR S19 OR S20 OR S21 OR S22 OR S23 (77,379)

S25 MH("Intervention Trials" OR "Double-Blind Studies" OR "Clinical Trials" OR "Single-Blind Studies" OR "Triple-Blind Studies" OR "Therapeutic Trials" OR "Randomized Controlled Trials" OR "Equivalence Trials" OR "Preventive Trials" OR "Nonrandomized Trials" OR "Systematic Review" OR "Scoping Review" OR "Meta Analysis") (359,087)

S26 PT ("Systematic Review" OR "Meta Analysis" OR "Clinical Trial" OR "Randomized Controlled Trial") (256,770)

S27 TI ( rct or cct or "random allocat*" or randomly or randomized or randomised or cluster-random* or "cluster random*" or "stepped wedge" or "clinical trial" or "controlled study" or "controlled trial" or "control group*" or blinding or blinded or masking or "double-blind*" or "single-blind*" or "systematic review*" or meta-analysis ) OR AB ( rct or cct or "random allocat*" or randomly or randomized or randomised or cluster-random* or "cluster random*" or "stepped wedge" or "clinical trial" or "controlled study" or "controlled trial" or "control group*" or blinding or blinded or masking or "double-blind*" or "single-blind*" or "systematic review*" or meta-analysis ) (445,897)

S28 TI (systematic* N2 (review* or overview*)) OR AB (systematic* N2 (review* or overview)) (92,940)

S29 TI (trial or trials) (127,342)

S30 S25 OR S26 OR S27 OR S28 OR S29 (622,240)

S31 S4 AND S7 AND S22 AND S8 (102)

**5. Embase**
Database coverage: 1980 to 2020 week 11
Interface/URL: OvidSP
Search date: 20-03-21
Number of records retrieved: 516
Search strategy:

| 1. pregnancy/ or Pregnant Woman/ or Mother/ or Maternal health/ (628186) |  |
| --- | --- |
| 2. (pregnan* or trimester* or mother* or matern* or expecting wom* or expecting mother* or expecting female* or expectant wom* or expectant mother* or expectant female* or antenatal* or prenatal* or pre-natal* or pre natal* or post-conception* or post conception* or postconception).ti,ab,kw. (969242) |  |
| 3. (gestat* and (women* or woman* or female* or mother* or matern*)).ti,ab,sh,kw. (247046) |  |
| 4. 1 or 2 or 3 (1173090) |  |
| 5. immunotherapy/ or exp immunization/ or exp vaccination/ or exp Vaccine/ (479214) |  |
| 6. (vaccine* or immunizat* or immunisat* or immunotherap* or inoculat*).ti,ab,kw. (607990) |  |
| 7. 5 or 6 (738610) |  |
| 8. exp low birth weight/ or Birth Weight/ or Fetus Weight/ or Small for date infant/ (119372) |  |
| 9. (lbw or vlbw or elbw or birth weight* or weight at birth or neonatal weight* or neonatal measure* or neonatal length* or newborn weight* or newborn measure* or newborn length* or low weight* or small for gestational age or small-for-gestational age or sga or fetal weight* or foetal weight* or fetal growth or foetal growth or fetal measure* or foetal measure* or fetal length* or foetal length* or birth length* or birth anthropometr* or birth measure* or infant measure* or head circumferen* or (weight for age adj3 birth) or (anthropometr* adj3 birth) or (small* adj3 (babies or neonat* or newborn*)) or (small adj3 gestational age)).ti,ab,kw. (127724) |  |
| 10. (iugr or fgr or ((fetal or foetal or in utero or intrauterine or intra-uterine) adj3 (growth restrict* or growth retard* or growth disturb* or growth abnormalit* or growth disorder* or growth trajector* or length trajector* or weight trajector*))).ti,ab,kw. (29929) |  |
| 11. exp prematurity/ or Premature Labor/ (134275) |  |
| 12. (prematurity or ((prematur* or preterm* or pre-term*) adj3 (babies or neonat* or newborn* or birth* or childbirth* or delivery or labor or labour or parturiti*))).ti,ab,kw. (106300) |  |
| 13. premature fetus membrane rupture/ or uterine cervix incompetence/ (11148) |  |
| 14. (premature ruptur* or Preterm ruptur* or Prelabor ruptur* or pre-labor ruptur* or Prelabour ruptur* or pre-labour ruptur* or PPROM or cervical incompetence or cervical insufficiency or cervical weakness).ti,ab,kw. (11041) |  |
| 15. Fetus Death/ or Fetus Mortality/ or Stillbirth/ (36231) |  |
| 16. (stillbirth* or still-birth* or still birth* or stillborn* or still-born* or still born* or ((fetal or foetal or antepartum or intrapartum or antenatal* or prenatal* or intrauterin* or intra-uterin* or in utero) adj3 (loss or death* or mortality or morbidity or demise))).ti,ab,kw. (46430) |  |
| 17. ((neonatal or birth) adj3 outcome*).ti,ab,kw. (29575) |  |
| 18. 8 or 9 or 10 or 11 or 12 or 13 or 14 or 15 or 16 or 17 (340292) |  |
| 19. exp clinical trial/ or clinical trials as topic/ or randomization/ or exp randomized controlled trial/ or randomized controlled trial as topic/ or meta analysis/ or Meta Analysis as Topic/ or systematic review/ or control group/ or double blind procedure/ or single blind procedure/ (1990528) |  |
| 20. (article or article in press or review).pt. (22954209) |  |
| 21. (rct or cct or random allocat* or randomly or randomized or randomised or cluster-random* or cluster random* or stepped wedge or clinical trial or controlled study or controlled trial or control group* or blinding or blinded or masking or double-blind* or single-blind* or systematic review* or meta-analysis or (systematic* adj2 (review* or overview*))).ti,ab,kw. or (trial or trials).ti. (2264411) |  |
| 22. 19 or 21 (3136906) |  |
| 23. 4 and 7 and 18 and 20 and 22 (605) |  |
| \| 24. \| Case study/ or abstract report/ or letter/ \| 1152858 \| \| --- \| --- \| --- \| \| 25 \| case report.ti,ab. \| 395299 \| \| 26 \| (conference abstract or conference paper or conference review).pt. \| 4494032 \| \| 27 \| 24 or 25 or 26 \| 5915748 \| \| 28 \| 23 not 27 \| 593 \| \| 29 \| 28 not ((exp animal/ or nonhuman/) not exp human/) \| 516 \| \|  \|  \|  \| |  |
|  |  |
|  |  |
|  |  |
|  |  |
|  |  |
|  |  |
|  |  |
|  |  |
