# Supplementary figures and images for: Modular literature review: a novel systematic search and review method to support priority setting in health policy and practice
Source: BMC Med Res Methodol. 2021 Nov 27;21:268. doi: 10.1186/s12874-021-01463-y (PMC8627616; doi:10.1186/s12874-021-01463-y)

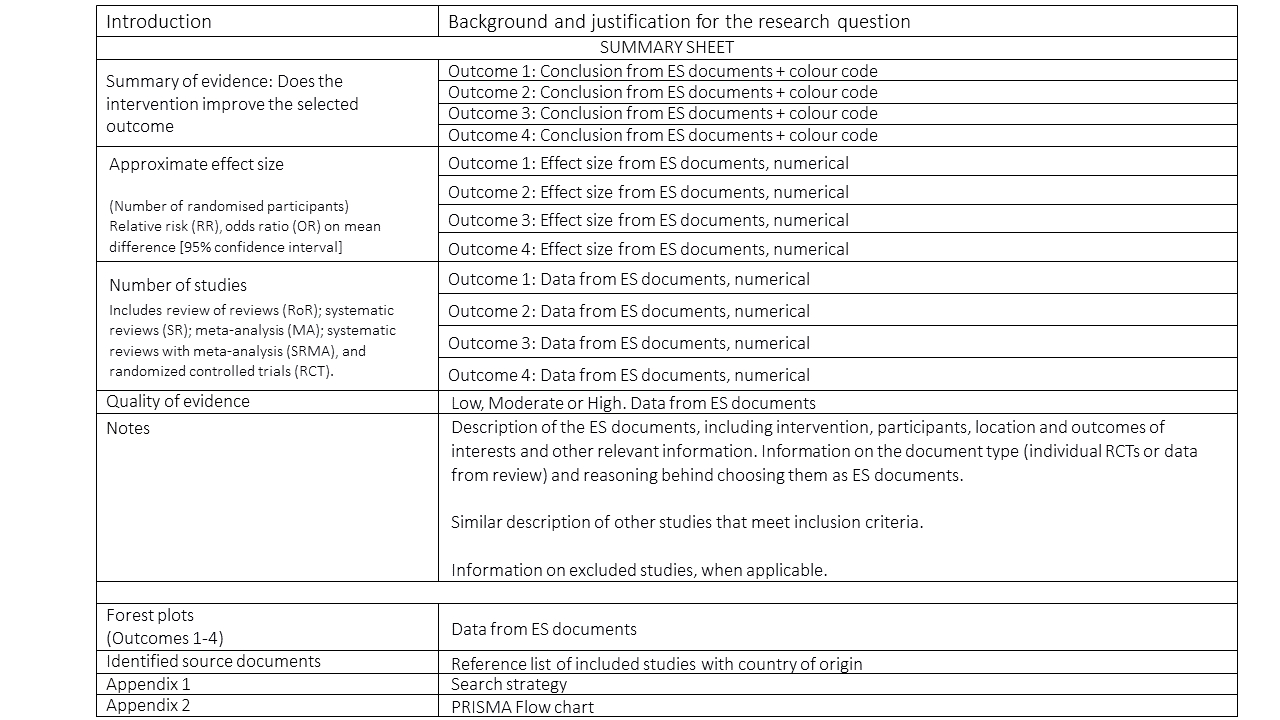

Supplement: Supplementary file 4 — Additional file 4. [file 12874_2021_1463_MOESM4_ESM.jpg]
